# Study of theThermo-/pH-Sensitivity of Stereo-Controlled Poly(*N*-isopropylacrylamide-co-IAM) Copolymers via RAFT Polymerization

**DOI:** 10.3390/polym10050512

**Published:** 2018-05-09

**Authors:** Syang-Peng Rwei, Whe-Yi Chiang, Tun-Fun Way, Huynh Nguyen Anh Tuan, Ya-Chin Chang

**Affiliations:** Institute of Organic and Polymeric Materials and Research and Development Center for Smart Textile Technology, National Taipei University of Technology, Taipei 106, Taiwan; wheyichiang@gmail.com (W.-Y.C.); tfway1951@gmail.com (T.-F.W.); tuanhna@hcmute.edu.vn (H.N.A.T.); yea.emptiness@gmail.com (Y.-C.C.)

**Keywords:** smart polymer, thermo- and pH-Sensitivity, *N*-isopropylacrylamide, itaconamic acid, UCST, LCST, soluble-insoluble-soluble transition, re-dissolved behavior

## Abstract

In this work, a smart copolymer, Poly(nipam-co-IAM) was synthesized by copolymerization of *N*-isopropylacrylamide (nipam) and itaconamic acid (IAM) through reversible addition-fragmentation chain-transfer (RAFT) polymerization. Poly(nipam-co-IAM) has been studied previously synthesized via radical polymerization without stereo-control, and this work used cumyl dithiobenzoate and Ytterbium(III) trifluoromethanesulfonate as RAFT and stereo-control agents, respectively. The stereo-control result in this work shows that tacticity affects the lower critical solution temperature (LCST) and/or the profile of phase separation of Poly(nipam-co-IAM). In the pH 7 and pH 10 buffer solutions, the P(nipam-co-IAM) copolymer solutions showed soluble–insoluble–soluble transitions, i.e., both LCST and upper critical solution temperature (UCST) transitions, which had not been found previously, and the insoluble to soluble transition (redissolved behavior) occurred at a relatively low temperature. The insoluble to soluble transition of P(nipam-co-IAM) in alkaline solution occurred at a temperature of less than 45 °C. However, the redissolved behavior of P(nipam-co-IAM) was found only in the pH 7 and pH 10 buffer solutions and this redissolved behavior was more prominent for the atactic copolymers than in the isotactic-rich ones. In addition, the LCST results under our experimental range of meso content did not show a significant difference between the isotactic-rich and the atactic P(nipam-co-IAM). Further study on the soluble-insoluble-soluble (S-I-S) transition and the application thereof for P(nipam-co-IAM) copolymers will be conducted.

## 1. Introduction

Poly *N*-isopropylacrylamide (Pnipam) is a thermal responsive polymer and Pnipam-based copolymers with the addition of another comonomer can become multiple stimuli-responsive copolymers, such as thermal and pH- dual stimuli-responsive polymers. These have the potential to be used in biomedical applications, such as controlled drug release [1,2,3,4,5,6,7,8,9,10,11,12,13,14,15,16,17,18]. In previous reports, Pnipam has shown a tacticity effect at its lower critical solution temperature (LCST) [19]. Isotactic-rich Pnipam (synthesized with stereo-control) has a lower LCST than atactic Pnipam (synthesized without stereo-control). On the other hand, it was previously reported that IAM (itaconamic acid) can be used as a comonomer to prepare P(nipam-co-IAM) copolymers with pH-sensitive segments [10]. P(nipam-co-IAM) was found to have a LCST but the tacticity effect of the copolymer has not previously been reported.

Aside from polymers with LCSTs, such as PNIPAM, PHPMA, and POEGMA [20,21], the presence of an upper critical solution temperature (UCST) in thermo-responsive polymers in aqueous solution is uncommon. Such polymers show an insoluble–to–soluble (I-S) transition when heated. Polymers, such as poly(acrylic acid) [22], poly(*N*-*tert*-butylacrylamide)-2,2,6,6-tetramethyl-1-piperdinyloxy nitroxide (PNTBA-TEMPO) block copolymer [23] have been reported to have UCSTs. The disruption of hydrogen bonds and ionic interactions [24] at a higher temperature may be attributed to the insoluble to soluble (I-S) transition. The nipam and acrylic acid (AA) copolymers have been of great interest in previous research because poly(acrylic acid) shows a UCST-type transition (insoluble to soluble; I-S transition) and Pnipam shows an LCST-type transition (soluble to insoluble; S-I transition) [22]. Therefore, the nipam/AA copolymer shows both LCST-type and UCST-type transitions, or the S-I-S transition, when the copolymer contains substantial fractions from both composition units. There are some polymers that show the S-I-S transition or display lower and upper critical solution temperatures. Reported examples include the nipam-AA copolymer [22], the AA-vinylacetamide copolymer [25], P(HPMAB) and P(PMAB-COOH) [26] where HPMAB is “dimethyl 3,3′-(((1-(2-hydroxy-3-(methacryloyloxy)propyl)-1H-1,2,3-triazol-4-yl)methyl)azanediyl) dipropanoate”. The nipam-AA copolymer contains 17 mol % or more AA units leading to the S-I-S transition, and the temperature range of this is 20~80 °C [22]. The thermal responsive HEMA-based polymers (2-hydroxyethyl methacrylate polymer) have been reported to show the S-I-S transition in the range of 30~90 °C [24]. Poly(ethylene oxide) (PEO) was reported to have the S-I-S transition, but the UCST-type transition appears at high temperatures (higher than 200 °C) and high pressures [27]. The phase behavior with the S-I-S transition is important scientifically and technologically because the conditions at which S-I-S transitions are found are not robust, and thus, a useful temperature range for tuning these transitions is still unknown and challenging. Therefore, the factors and the mechanisms of the S-I-S transitions are drawing great interest.

Controlled-living radical polymerization, especially reversible addition-fragmentation chain-transfer (RAFT) polymerization, provides the benefit of being able to readily synthesize polymers with predetermined molecular structures and narrow molecular weight distributions [28,29,30,31]. In addition, RAFT polymerization can be applied to bulk, solution, emulsion or suspension polymerizations. RAFT polymerization can be implemented by introducing a chain transfer agent, known as a RAFT agent, to a conventional radical polymerization reaction. Thiocarbonylthio compounds, including dithoester and trithiocarbonate, are commonly used as RAFT agents. The addition of Lewis acid during polymerization can affect the stereochemistry of the polymers [19,29,30,31].

The copolymers in the current work were synthesized using nipam as a major monomer to give thermo-sensitive or thermo-responsive segments and IAM (itaconamic acid) [10,32] was used as a second monomer to give pH-sensitive segments. The utilization of reversible addition-fragmentation chain-transfer (RAFT) polymerization was intended to decrease the polydispersity (PDI) or narrow the molecular weight distribution of the synthesized copolymer. In addition, the tacticity was studied by addition of Lewis acid (Ytterbium(III) trifluoromethanesulfonate; Y(OTf)_3_). The properties of P(nipam-co-IAM) with and without stereo-control were investigated.

## 2. Experimental

### 2.1. Materials

*N*-isopropylacrylamide (nipam; C_6_H_11_NO) obtained from Aldrich Chemical Corp. (St. Louis, MO, USA) was purified twice through recrystallization from n-hexane. Itaconamic acid (IAM; C_5_H_7_NO_3_ also called “4-amino-2-methylene-4-oxobutanoic acid”) was prepared according to the method disclosed in US patent publication No. 2013/0172490 [32]. 2,2′-Azobis(isobutyronitrile) (AIBN) (purity 98%), cumyl dithiobenzoate (CDB), and *N*,*N*-dimethylformamide (DMF) (purity 99.8%) were obtained from Aldrich Chemical Corp. (St. Louis, MO, USA) and used as received without any further purification. Ytterbium(III) trifluoromethanesulfonate (Y(OTf)_3_) was obtained from TCI Co. (Tokyo, Japan). The buffer solution of pH 4, obtained from J. T. Baker Chemical Corp. (Center Valley, PA, USA), was prepared from potassium hydrogen phthalate and sodium benzoate; the buffer solution of pH 5, obtained from Alfa Aesar Co. (Tewksbury, MA, USA) and the buffer solution of pH 6 obtained from Sigma Aldrich Corp. (St. Louis, MO, USA), were prepared from citric acid and sodium hydroxide; the buffer solution of pH 7, obtained from J. T. Baker Chemical Corp. (Center Valley, PA, USA), was prepared from potassium dihydrogen phosphate, disodium hydrogen phosphate and sodium benzoate; the buffer solution of pH 8, obtained from Sigma Aldrich, was prepared from sodium tetraborate and hydrochloric acid; and the buffer solution of pH 10, obtained from J. T. Baker Chemical Corp., was prepared from borate solution.

### 2.2. Synthesis of Stereo-Controlled Poly(nipam-co-IAM) Copolymers (I-12Y and I-8Y)

**Scheme 1:**
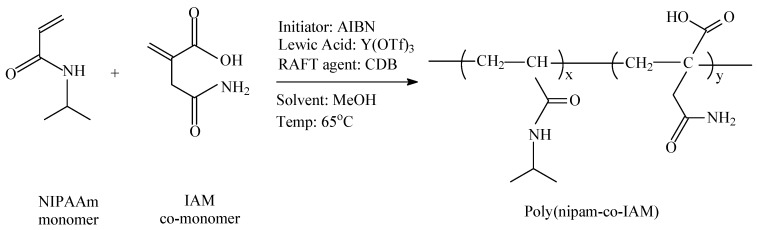
RAFT polymerization of *N*-isopropylacrylamide (NIPAAm monomer) and itaconamic acid (IAM co-monomer).

Poly(nipam-co-IAM) copolymers were synthesized by reversible addition-fragmentation chain-transfer (RAFT) polymerization, as shown in Scheme 1. Nipam (14.6 mmol) and itaconamic acid (IAM) (with a molar ratio of nipam/IAM = 100/12 or 100/8; that is, 1.75 or 1.17 mmol) were dissolved in methanol (5 mL) in a flask and 2,2′-azobis(isobutyronitrile) (AIBN; 0.037 mmol) as the initiator, Ytterbium(III) trifluoromethanesulfonate (Y(OTf)_3_; 0.031 mmol) as a Lewis acid and cumyl dithiobenzoate as a RAFT agent (CDB; 0.12 mmol) were added to the flask. The mixture was stirred for 10 min until it became homogeneous. The reaction flask was cooled by a 5 °C water bath, vacuumed and nitrogen-purged before the polymerization reaction. Then, the flask was kept at about 65 °C in the nitrogen atmosphere for polymerization for 24 h. After the reaction was completed, the reaction mixture was cooled in the air. Water was added to dissolve the reaction product, and the obtained solution was placed in a dialysis membrane with molecular weight cut off of 10,000 g/mol for 3 days (refreshed every half day). Then, the reaction product was filtered by a 0.4 μm filter and stored in a sample vial to be frozen in a refrigerator. Finally, the obtained solution was freeze-dried at −50 °C to completely remove water. White solid powders were obtained as samples I-12Y and I-8Y for nipam/IAM = 100/12 and 100/8, respectively.

### 2.3. Synthesis of Stereo-Controlled Poly(nipam) Homopolymer (P-Y)

The Pnipam homopolymer was also synthesized by RAFT polymerization as a comparison to the Poly(nipam-co-IAM) copolymers in this study where the process was the same as the above method except that IAM was not added. The obtained Pnipam homopolymer was labeled as P-Y.

### 2.4. Synthesis of Non-Stereo-Controlled Poly(nipam-co-IAM) Copolymers (I-12 noLA and I-8 noLA)

Polymerization without addition of the Lewis acid was conducted as described in the paragraph regarding the synthesis of the stereo-controlled P(nipam-co-IAM) copolymers.

### 2.5. Synthesis of Non-Stereo-Controlled Pnipam Homopolymer (P_noLA)

The polymerization without addition of the Lewis acid was conducted as described in the paragraph about the synthesis of the stereo-controlled Pnipam homopolymer.

NMR spectra were measured using a Bruker Advance 300 MHz NMR spectrometer (Billerica, MA, USA) by weighing 10 mg of a test sample and dissolving in 1 mL of DMSO-d_6_ placed in a standard 507-HP NMR test tube.

FT-IR spectra were measured by Perkin Elmer Spectrum RXI FTIR (Waltham, MA, USA) within 4000–400 cm^−1^ with a resolution of 4.00 cm^−1^.

A Viscotek GPC system from Malvern Ltd. (Malvern, UK) was used to measure the weight average molecular weight (M_w_), number average molecular weight (M_n_) and polydispersity index (PDI) of the linear copolymer, p(nipam-co-IAM) and the homopolymer, Pnipam. The test samples were prepared by weighing about 15 mg of test material and dissolving this in 10 mL of DMF with a 300 × 8 mm column and a flow rate of 1 mL/min. The temperature of the column was set to 60 °C, the temperature of the detector was set to 60 °C, and the injection quantity of the test sample and the standard was 150 μL each.

The hydrodynamic radii of the linear copolymer P(nipam-co-IAM) and homopolymer Pnipam were measured by dynamic light scattering (DLS, Malvern 1000 HSA was used, Malvern, UK). The 0.5 wt % aqueous solutions of samples were measured at 25 °C.

The phase separation profiles and temperatures were determined from the transmittance of the sample containing the Pnipam homopolymer or P(nipam-co-IAM) copolymers as a function of temperature using a laser transmittance meter (LASOS LGK 7628). The LCST was determined when the transmittance of the solution was 50%.

The test samples containing Pnipam homopolymer and P(nipam-co-IAM) copolymers were prepared by adding Pnipam homopolymer or P(nipam-co-IAM) copolymers (P-Y, I-8Y, I-12Y, P-noLA, I-8 noLA, I-12 noLA) to water in a 1 mL vial to obtain samples with a 1 wt % concentration. The samples containing Pnipam homopolymer and P(nipam-co-IAM) copolymers in the buffer solutions with different pH values (4~10) were prepared by adding solid samples in the buffer solutions (pH = 4~10) to get 1 wt % polymer solutions which were used to study the phase separation behavior at different pH values.

## 3. Results and Discussion

### 3.1. ^1^H NMR Analysis

**Figure 1:**
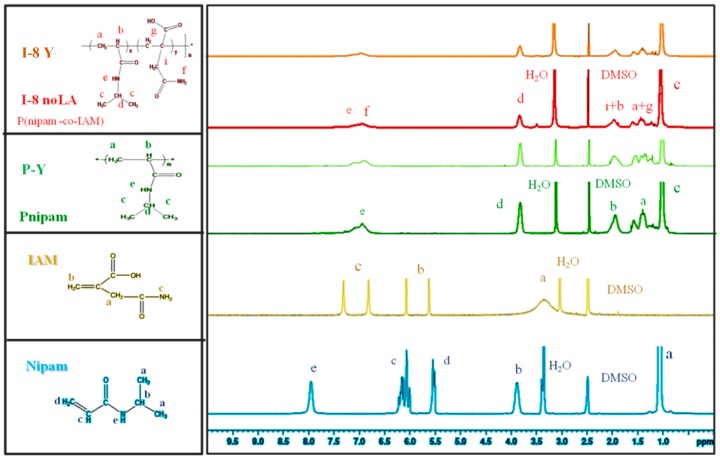
^1^H NMR spectra of the nipam monomer, the IAM comonomer, Pnipam and P(nipam-co-IAM).

Figure 1 shows the ^1^H NMR spectra of the nipam monomer, the IAM comonomer, Pnipam and P(nipam-co-IAM). The assignments are shown, correspondingly, on the left-hand side of Figure 1, indicating successful synthesis of Pnipam and P(nipam-co-IAM). For example, the peaks from Nipam at δ = 5.5 ppm and δ = 6.1 ppm (bands “c” and “d”) and from IAM at δ = 5.6 ppm and δ = 6.0 ppm (band “b”) are absent in the spectra of Pnipam and P(nipam-co-IAM).

**Figure 2:**
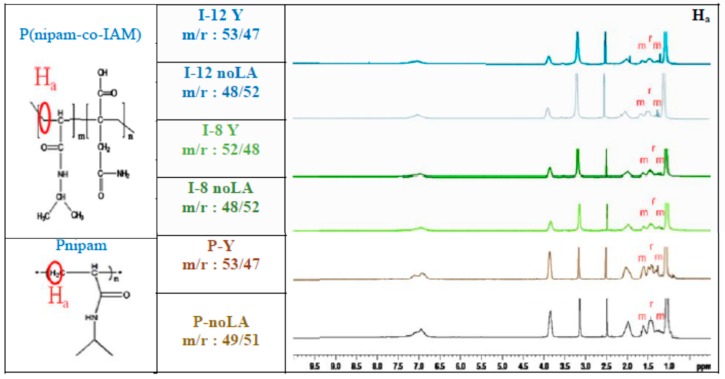
^1^H NMR spectra of Pnipam and poly(nipam-co-IAM) at 60 °C obtained with a 600 MHz NMR spectrometer.

Previous reports have indicated that NMR spectra at elevated temperatures can reveal more detailed characteristics of a functional group. Therefore, in this work, the tacticity levels of Pnipam and P(nipam-co-IAM) were measured using ^1^H NMR spectra at 60 °C [33,34]. Figure 2 shows the ^1^H NMR spectra of Pnipam and P(nipam-co-IAM) which were conducted by a 600 MHz NMR spectrometer at 60 °C. As shown in Figure 2, the peaks (m, r, m) between δ = 1.1~1.8 ppm are attributed to the splitting of the methylene group (–C**H**_2_CH–; H_a_). The tacticity can be calculated from the following equations:

$$\mathrm{meso}\;\mathrm{diad}\;\mathrm{content}\;\left(m\right)\;\%=\frac{\mathrm{meso}\;\mathrm{peak}\;\mathrm{integrated}\;\mathrm{value}}{\left(\mathrm{meso}+\mathrm{racemo}\right)\;\mathrm{peak}\;\mathrm{integrated}\;\mathrm{value}}\times 100\%$$

and

$$\mathrm{racemo}\;\mathrm{diad}\;\mathrm{content}\;\left(r\right)\;\%=\frac{\mathrm{racemo}\;\mathrm{peak}\;\mathrm{integrated}\;\mathrm{value}}{\left(\mathrm{meso}+\mathrm{racemo}\right)\;\mathrm{peak}\;\mathrm{integrated}\;\mathrm{value}}\times 100\%$$

From the spectra of Pnipam with and without Lewis acid (sample “P-Y” and “P-noLA”), shown in Figure 2, the content of meso diads was calculated to be 53% for P-Y and 49% for P-noLA, which is consistent with a previous report by Nishi et al. [35]. On the other hand, for P(nipam-co-IAM), the stereo-controlled copolymers (I-8Y and I-12Y) had meso contents (m) of 52~53% and the non-stereo-controlled (atactic) copolymers (I-8 noLA and I-12 noLA) had meso contents (m) of about 48%, in the presence of 6 × 10^−3^ M of Lewis acid (Y(OTf)_3_). The meso diad content of the stereo-controlled copolymers was smaller than that of the homopolymer, which may be because IAM is more bulky and therefore blocks the coordination bonding of ytterbium to double bonds. In addition, bidentate IAM can also form coordination bonding with ytterbium to decrease the amount of coordination bonding of ytterbium to double bonds.

### 3.2. FTIR Analysis

**Figure 3:**
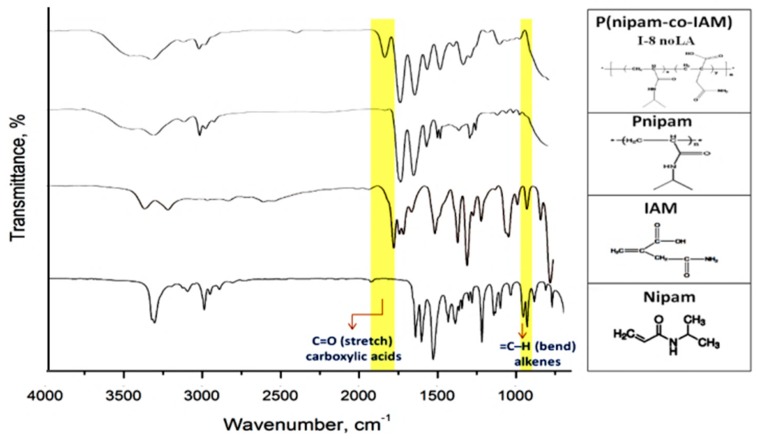
FTIR spectra of nipam, IAM monomers, atactic Pnipam (P-noLA) and P(nipam-co-IAM) (I-8 noLA).

**Figure 4:**
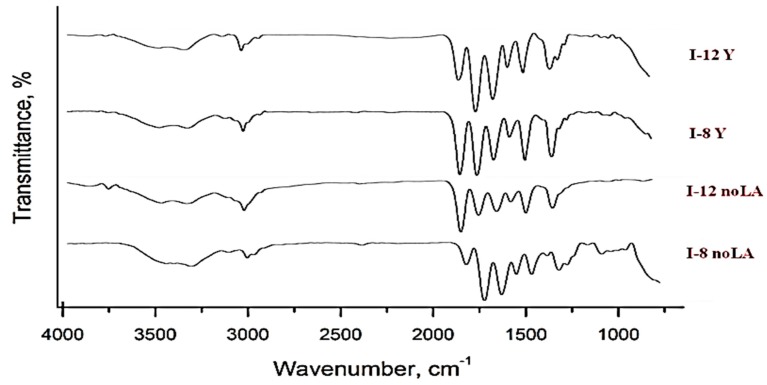
FTIR spectra of P(nipam-co-IAM) with and without stereo-control.

Figure 3 shows the FTIR spectra of nipam, IAM monomers and Pnipam (P-noLA), P(nipam-co-IAM) (I-8 noLA) without stereo-control. Figure 4 shows the FTIR spectra of P(nipam-co-IAM) with and without stereo-control.

In the spectrum of Nipam, the amide C=O stretching peak is shown at about 1650 cm^−1^; the amine N–H bending peak is shown at about 1550 cm^−1^; and the amine N–H stretching peak is shown at 3600~3200 cm^−1^. After polymerization, the spectrum of Pnipam shows an absence of the peak at about 960 cm^−1^, indicating that C=C formed C–C in Pnipam with the RAFT method.

In the spectrum of P(nipam-co-IAM) (I-8 noLA) in Figure 3, the peak at about 1700 cm^−1^ is attributed to the C=O stretch of the carboxylic group of IAM, and the peak at about 3600~3200 cm^−1^ is attributed to the O–H of the carboxylic group of IAM, which overlaps with the amine N–H stretching peak. The FTIR spectra also confirm the successful synthesis of the P(nipam-co-IAM) copolymers.

However, as shown in Figure 4, the tacticity effects of Pnipam and P(nipam-co-IAM) were not found in the FTIR spectra which is consistent with the report from Hasegawa et al. [36] showing that the IR characteristic peaks of IR-ATR (attenuated total reflectance) spectra can only be shifted slightly.

### 3.3. Gel Permeation Chromatography (GPC) Analysis

**Scheme 2:**
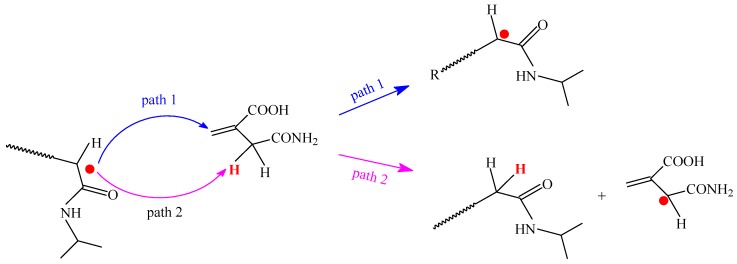
The mechanism of the effect of IAM comonomer on copolymerization.

The number average molecular weight (M_n_) and PDI (M_w_/M_n_) of Pnipam and P(nipam-co-IAM) calculated from the GPC results together with particle diameters are shown in Table 1. As shown in Table 1, the number average molecular weights (M_n_) of P(nipam-co-IAM) synthesized by RAFT were controlled within about 30,000~40,100 and the polydispersity index (PDI; M_w_/M_n_) of P(nipam-co-IAM) synthesized by RAFT was about 2.0, similar to that produced by general radical polymerization [10]. RAFT polymerization was expected to narrow the molecular weight distribution, i.e., the polymer synthesized by RAFT should have a low PDI value. Each individual polymer shown in Table 1 has a lower PDI value when using the Lewis acid agent, for example, P_noLA (PDI = 1.48) versus PY (PDI = 1.33) and I_8 noLA (PDI = 1.91) versus I_8Y (PDI = 1.86). However, as the molar fraction of IAM in P(nipam-co-IAM) increased, PDI increased no matter whether RAFT agent was used or not. This phenomenon may be due to the influence of the IAM comonomer to the copolymerization and is illustrated in Scheme 2. As shown in Scheme 2, the reactive radical may react with IAM via path 1 or path 2. The reaction via path 1 results in chain growth reaction, as usual, which grows the polymer chain (larger molecular weight), while the reaction via path 2 results in the chain-transfer reaction and chain termination, giving a lower molecular weight. Since IAM has both allyl active α-hydrogen and carbonyl active α-hydrogen, this active α-hydrogen can accelerate and strengthen the chain-transfer reaction. Therefore, these two reaction pathways of IAM broaden the molecular weight distribution. This result is also consistent with the report of Tada et al. [37] for meso diad-rich Pnipam which was prepared by RAFT polymerization. In another study, Ray et al. reported that the PDI of isotactic Poly(nipam-b-St) diblock copolymer produced using RAFT polymerization was also higher than that of an atactic one (PDI 1.90 for isotactic copolymer compared to PDI 1.58 for atactic copolymer). This phenomenon was attributed to the chain termination of some chains, which occurs during polymerization, or was due to the inclusion of some homopolymers [28].

### 3.4. LCST Analysis

The phase separation behavior of P(nipam-co-IAM) together with Pnipam as the reference was investigated using 1 wt % aqueous solutions which were prepared by dissolving Pnipam and P(nipam-co-IAM) in buffer solutions of pH 4~10. The pH sensitivity of the LCSTs was also investigated using the buffer solutions. It should be noted that the aqueous solution of P(nipam-co-IAM) in deionized water quickly became acidic and has a pH value of less than 7 because of the –COOH groups of the IAM composition units. Therefore, the LCST of the sample dissolved in deionized water was not reported. In our previous report [10], the LCST value at pH 7 was from the sample dissolved in deionized water and thus, was not the LCST of Pnipam or P(nipam-co-IAM) in the pH 7 buffer solution.

**Figure 5:**
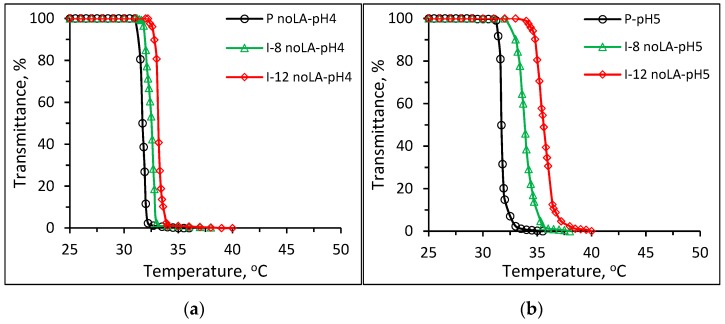

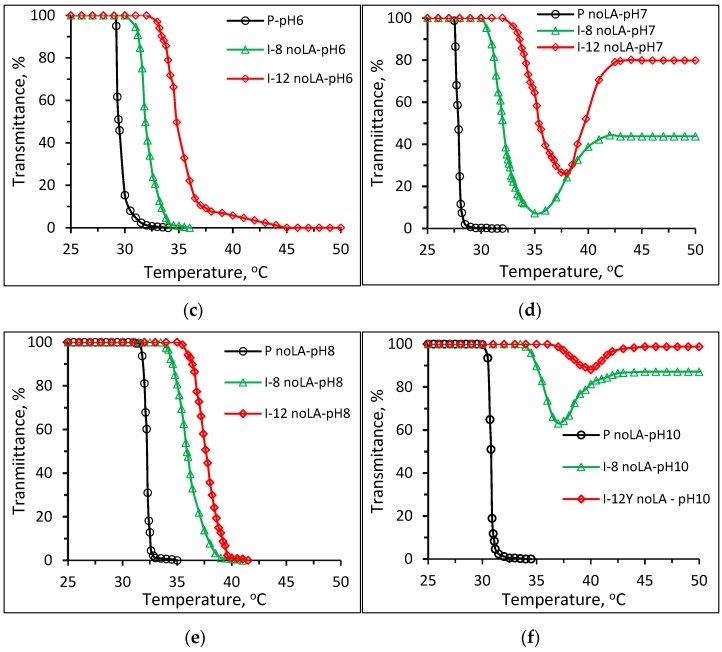
Plots of transmittance vs. temperature of 1 wt % aqueous solutions of Pnipam and P(nipam-co-IAM) synthesized without stereo-control in buffer solutions of (**a**) pH = 4, (**b**) pH = 5, (**c**) pH = 6, (**d**) pH = 7, (**e**) pH = 8 and (**f**) pH = 10.

Figure 5 shows plots of transmittance vs. temperature of 1 wt % aqueous solutions of Pnipam and P(nipam-co-IAM) synthesized without stereo-control in buffer solutions of (a) pH 4, (b) pH 5, (c) pH 6, (d) pH 7, (e) pH 8 and (f) pH 10. As shown in Figure 5a–c in the acidic buffer solutions (pH 4~6), the solutions of Pnipam and P(nipam-co-IAM) show the LCST-type transition, i.e., soluble to insoluble transition as the temperature increases. Figure 5d,f both show the soluble–insoluble–soluble transition (redissolution) in the heating range of 25~50 °C for samples I-8 and I-12 noLA (without stereo-control) at pH 7 and 10.

**Figure 6:**
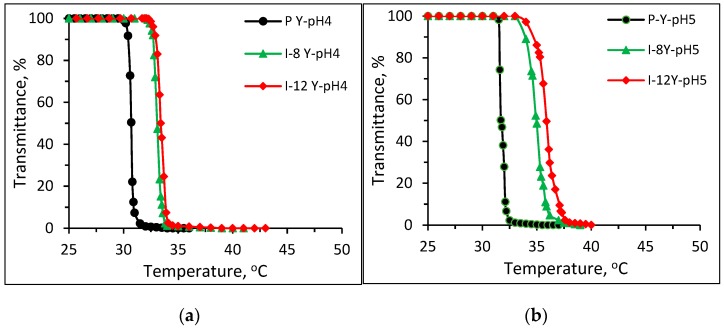

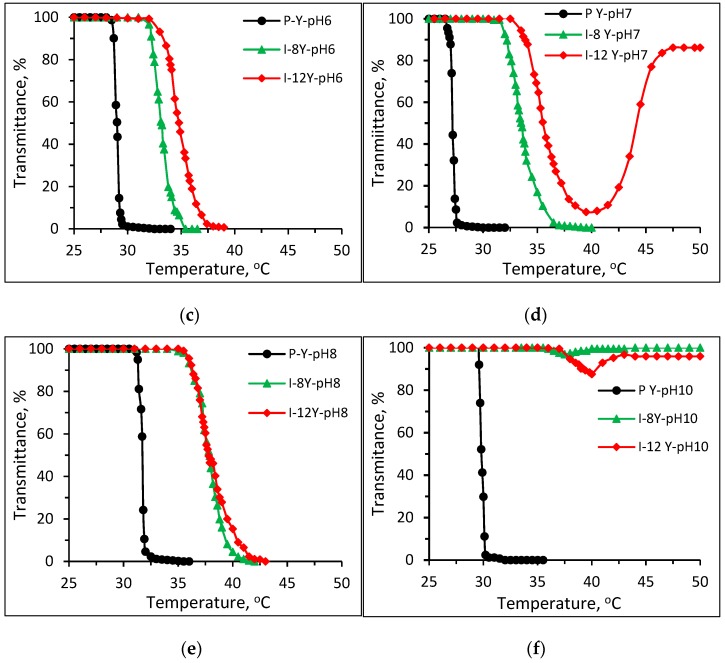
Plots of transmittance vs. temperature of 1 wt % aqueous solutions of Pnipam and P(nipam-co-IAM) synthesized with stereo-control in buffer solutions of (**a**) pH = 4, (**b**) pH = 5, (**c**) pH = 6, (**d**) pH = 7, (**e**) pH = 8 and (**f**) pH = 10.

On the other hand, Figure 6 shows plots of transmittance vs. temperature of 1 wt % aqueous solutions of Pnipam and P(nipam-co-IAM) synthesized with stereo-control in buffer solutions of (a) pH 4, (b) pH 5, (c) pH 6, (d) pH 7, (e) pH 8 and (f) pH 10. As shown in Figure 6a–c in the acidic buffer solutions (pH 4~6), Pnipam and P(nipam-co-IAM) also show the typical LCST transition which is similar to Figure 5a–c. Figure 6d,f both show the soluble–insoluble–soluble (S-I-S) transition (redissolution) in the heating range of 25~50 °C for samples I-8 and I-12 Y (with stereo-control) at pH 7 and 10.

The presence of the soluble–insoluble–soluble transition in a narrow temperature range was rare and was found to be present only for samples I-8 and I-12, synthesized both with and without stereo-control. The phenomenon is more prominent for atactic copolymers. The soluble–insoluble transition by heating gives a LCST, and then the insoluble–soluble transition upon further heating (redissolved at the higher temperature) gives a UCST. This may be due to the combined effect of the entropy of solvation and the enthalpy of the formation of intra- and inter-chain hydrogen bonds, causing copolymers to have the soluble–insoluble–soluble transition occur at very specific conditions [22,25,26].

**Figure 7:**
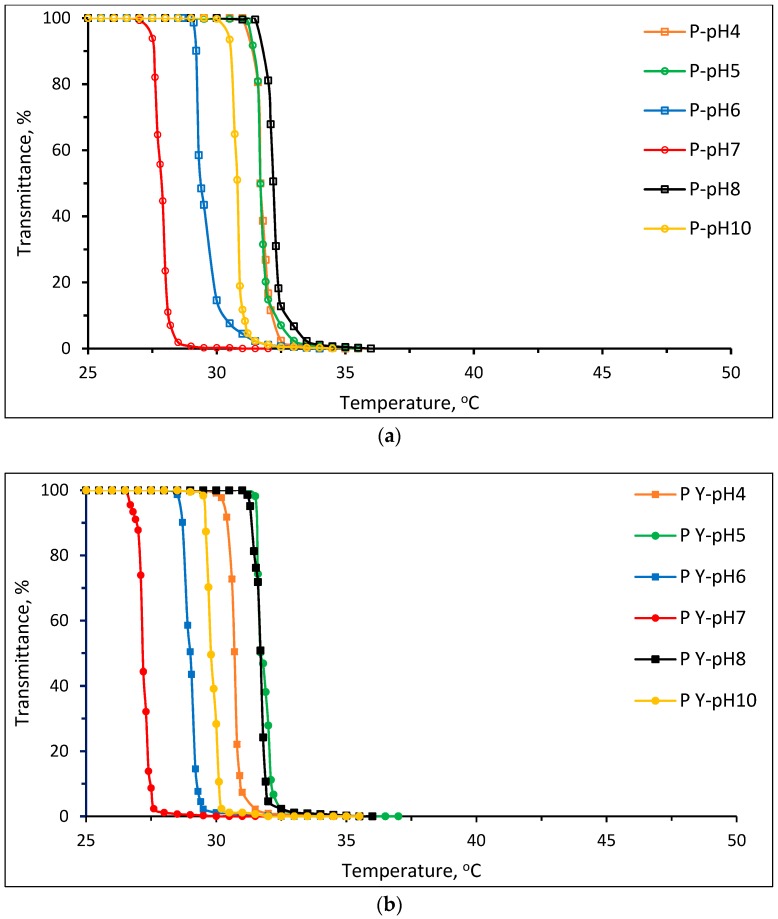
Plots of transmittance vs. temperature of 1 wt % aqueous solutions of Pnipam in buffer solutions of pH = 4~10 synthesized (**a**) without and (**b**) with stereo-control.

**Figure 8:**
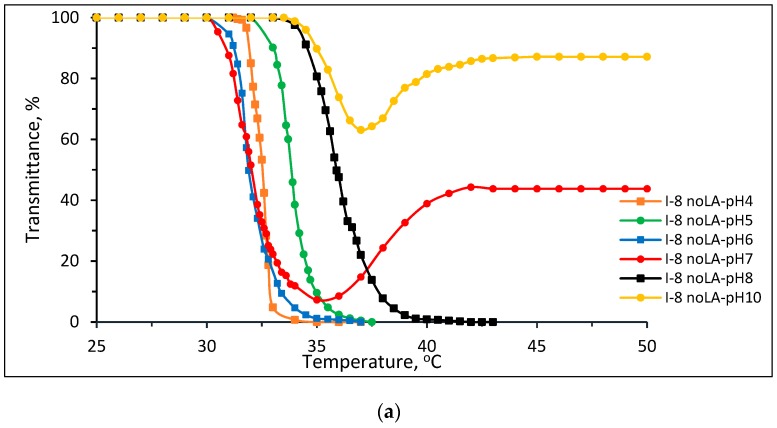

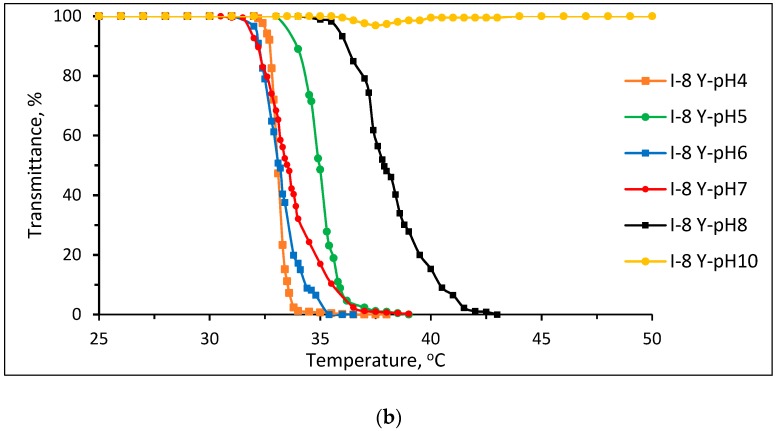
Plots of transmittance vs. temperature of 1 wt % aqueous solutions of P(nipam-co-IAM) (I-8) in buffer solutions of pH = 4~10 (**a**) without and (**b**) with stereo-control.

The stereo-control effect was investigated for Pnipam (Figure 7) and P(nipam-co-IAM) (sample I-8, Figure 8). Figure 7 shows plots of transmittance vs. temperature of 1 wt % aqueous solutions of Pnipam in buffer solutions of pH 4~10 synthesized (a) without and (b) with stereo-control. Figure 8 shows plots of transmittance vs. temperature of 1 wt % aqueous solutions of P(nipam-co-IAM) (I-8) in buffer solutions of pH 4~10 (a) without and (b) with stereo-control.

**Figure 9:**
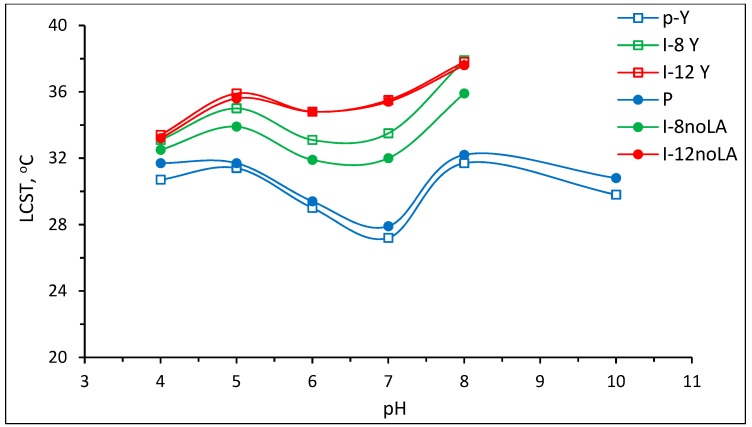
Determined lower critical solution temperature (LCSTs) (°C) of Pnipam and P(nipam-co-IAM) in various buffer solutions of pH = 4~10.

The LCSTs of Pnipam synthesized with and without stereo-control were 30.7 and 31.7 °C, respectively, in the pH 4 buffer solution and 29.8 and 30.8 °C, respectively, in the pH 10 buffer solution. The isotactic-rich Pnipam (synthesized with stereo-control) had a lower LCST than the atactic Pnipam (synthesized without stereo-control). The LCST result of the isotactic-rich Pnipam is consistent with the finding from a previous report [19]. Ray et al. [19] reported that the phase separation temperature decreased with an increase in tacticity. Apart from the LCSTs at pH 6 and 7, the LCST of Pnipam without stereo-control showed no pH sensitivity, i.e., the difference of LCSTs was less than ±1. It should be noted that the LCSTs and UCSTs can vary for different pH values, for example, the polymers at pH 6.4, 6.8, 7.4 and 7.8 from table 2 in the report [26]. Figure 9 summarizes the determined LCSTs (°C) of Pnipam and P(nipam-co-IAM) in various buffer solutions of pH 4~10 where the standard deviation is about ± 0.2 °C. As shown in Figure 9, the determined LCSTs showed non-systematic changes for both Pnipam and P(nipam-co-IAM). However, except for the LCSTs at pH 6 and 7, Pnipam showed no pH sensitivity, which was also shown in previous reports [10]. P(nipam-co-IAM) showed pH sensitivity. The anomaly of the LCSTs at pH 7 or around 7 was brought to attention for the first time and further study should be conducted. The abnormal results of the determined LCSTs may be due to various factors, such as the occurrence of the S-I-S transition to vary the determination of LCSTs, the large distribution of the molecular weights, polymerization methods and so forth. On the other hand, the data shown in Figure 9 indicates that P(nipam-co-IAM) still showed pH sensitivity because the LCST values at pH 4 were smaller than those at pH 8. In addition, the LCST of the copolymer increased with an increase in the IAM molar fraction.

The results of P(nipam-co-IAM) (I-8 Y) produced with stereo-control is shown in Figure 8b, and those of P(nipam-co-IAM) (I-8 Y) produced withno stereo-control are shown in Figure 8a. In the buffer solutions of pH 4~8, the isotactic-rich P(nipam-co-IAM) (I-8 Y) did not show the S-I-S transition, but in the pH 10 buffer solution, the transmittance dropped by only about 5% at about 37.5 °C. The atactic P(nipam-co-IAM) (I-8 noLA) in the pH 7 and pH 10 buffer solutions showed the S-I-S transition in the heating range of 25~50 °C. The isotactic-rich P(nipam-co-IAM) (I-8 Y) had a relatively smaller change in LCST than the atactic P(nipam-co-IAM) (I-8 noLA). As shown in Figure 8a, the atactic P(nipam-co-IAM) (I-8 noLA) has a useful pH-tunable thermo-responsive property; in particular, it is able to undergo S-I-S transition within the temperature range of 25~50 °C, giving it a broad range of applications, such as a drug carrier or separation medium.

**Figure 10:**
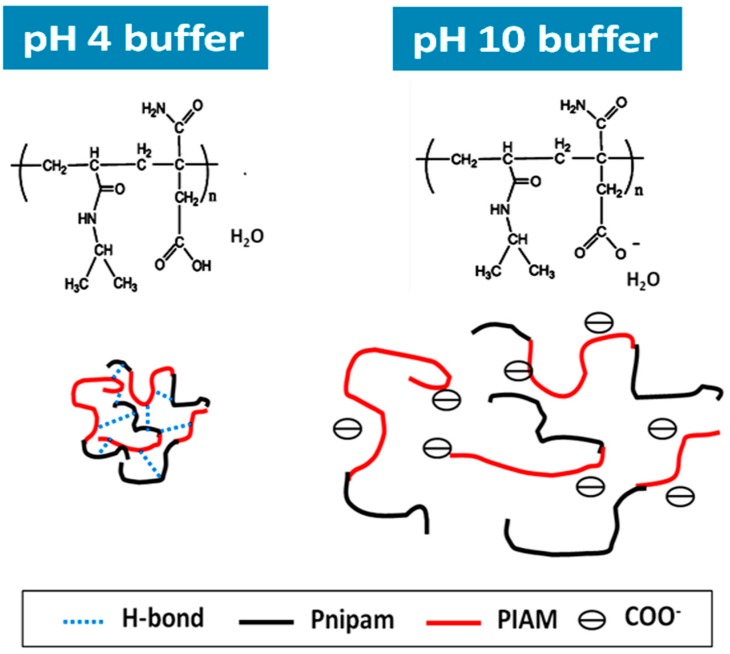
Proposed structures of P(nipam-co-IAM) in the buffer solutions with pH = 4 and 10.

The proposed structures of P(nipam-co-IAM) in the buffer solutions of pH 4 and 10 are shown in Figure 10. At a low pH value (acidic environment), the carboxyl groups are more likely to exist in the form of –COOH, while the carboxyl groups exist in the form of –COO^−^ at a high pH value or in an alkaline enviorment. That is, at a low pH value, less carboxyl groups from IAM units in the copolymer are charged, leading to the formation of hydrogen bonds between the COOH moiety from IAM and the NHCO moiety from nipam being enhanced. This would reduce the water solubility of the copolymer so that the LCST is lowered. In the high pH environment, the copolymer has more carboxyl groups from IAM units being charged to become swelled, leading to the enhancement of the formation of hydrogen bonds between polymer chain-segments and water. Thus, the LCST is higher at a high pH value and the trend is more significant for copolymers with a higher molar ratio of IAM to nipam.

In a previous study by Han et al. [38], the insoluble to soluble transition (UCST) that appears at higher temperatures may be attributed to specific interactions such as hydrogen bonds or ionic interactions being disrupted by free hydrophilic functional groups. Further study should be conducted for P(nipam-co-IAM) copolymers. As the temperature was raised to more than 40 °C, the redissolved behavior (insoluble to soluble transition) appeared. P(nipam-co-IAM) (I-8 noLA, I-12 noLA) showed the redissolved behavior but P(nipam-co-IAM) (I-12 noLA) was not significant, because the redissolved behavior seemed to highly depend on the IAM molar fraction of the copolymer, i.e., polymer composition.

The isotactic-rich P(nipam-co-IAM) showed less significant redissolving behavior than the atactic version, as shown in Figure 6 and Figure 8b, which may be because the polymer chains of the isotactic-rich P(nipam-co-IAM) are highly oriented, compared to the atactic P(nipam-co-IAM).

As shown in Figure 6, Figure 7, Figure 8 and Figure 9, P(nipam-co-IAM) showed pH sensitivity, and the phase separation behavior of P(nipam-co-IAM) was affected by polymer composition, ionic strength, pH, and tacticity. The conditions at which the S-I-S transition of P(nipam-co-IAM) copolymer was found were quite unpredictable and thus, further investigation of the redissolved behavior (both S-I and S-I transitions in a narrow range or S-I-S transition) is needed to be conducted using IAM as a comonomer.

**Figure 11:**
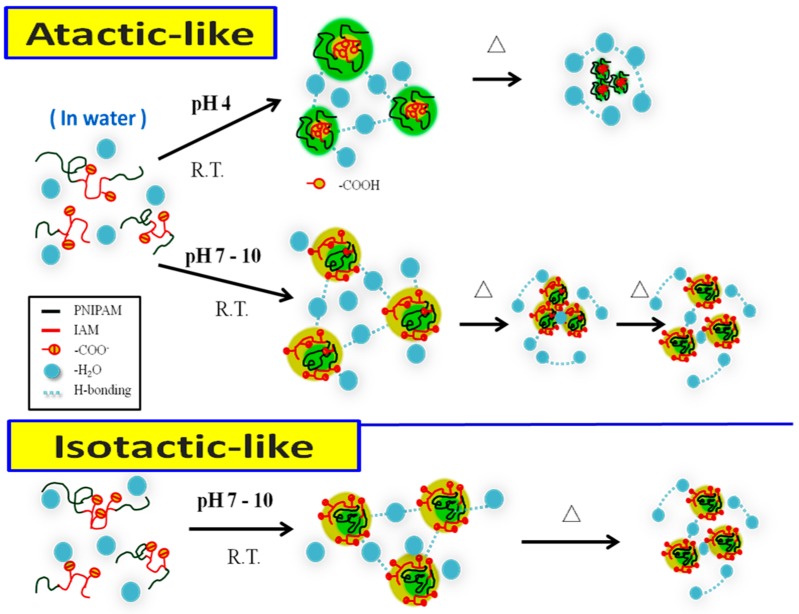
Proposed phase separation mechanism of 1 wt % aqueous solution of the atactic-like and the isotactic-like P(nipam-co-IAM) in the buffer solutions.

Figure 11 shows the proposed phase separation mechanism of 1 wt % aqueous solution of the atactic-like and the isotactic-like P(nipam-co-IAM) in the buffer solutions. As shown in Figure 11, micelle formation is proposed to occur in the alkaline solution producing –COO^−^ moieties (marked in red in the figure) of IAM segments which face outwards. The copolymer, I-12, which has a higher IAM molar fraction than I-8 also showed the redissolved behavior in the pH 7 and pH 10 buffer solutions but not in the pH 8 solution, which may indicate that the pH effect on redissolved behavior is difficult to predict due to delicate balance of ionic strength in the solutions. The formation of micelles may occur only for P(nipam-co-IAM) polymers with a specific range of IAM content at a specific pH value because P(nipam-co-IAM) is not synthesized as a block polymer in the current work. Therefore, micelle formation may only occur under special conditions.

## 4. Conclusions

In the current work, the thermal and pH dual responsive copolymers P(nipam-co-IAM) were prepared by reversible addition-fragmentation chain-transfer (RAFT) polymerization, with or without stereo-control. The tacticity effect on the thermal and pH responses of P(nipam-co-IAM) were investigated. In addition, the insoluble to soluble (UCST-type) transition of P(nipam-co-IAM) was found under very specific conditions.

The stereo-control result in this work shows that tacticity affects the lower critical solution temperature (LCST) and/or the profile of phase separation of Pniapm and Poly(NIPAM-co-IAM). The isotactic-rich Pnipam (synthesized with stereo-control) had a lower LCST (25.1 °C) than the atactic Pnipam (synthesized without stereo-control). Although the resulting polymers showed low meso content, the stereo-control experiment conducted in this work only tried to show the trends of the properties of the isotactic-rich polymers. In cases where IAM/nipam was equal to 8/100, the isotactic-rich P(nipam-co-IAM) had a slightly higher LCST than the atactic one. The LCST of the isotactic-rich copolymer increased with an increase in the IAM molar fraction. The LCST of P(nipam-co-IAM) (I-8 noLA) shifted from low to high temperatures as the pH value of the solution became higher. In the pH 7 and pH 10 buffer solutions, the P(nipam-co-IAM) copolymer solution showed the soluble–insoluble–soluble transitions, i.e., both LCST- and UCST-transitions, and the insoluble to soluble transition occurred at a relatively low temperature. The insoluble to soluble (UCST-type) transition of P(nipam-co-IAM) in the alkaline solutions occurred at temperatures below 45 °C. The LCST results in our experimental meso content range did not show a significant difference between isotactic-rich and atactic P(nipam-co-IAM).

The redissolved behavior of P(nipam-co-IAM) was found only in the pH 7 and pH 10 buffer solutions, and the re-dissolved behavior was more prominent for the atactic copolymers than for the isotactic-rich ones. Whether or not the S-I-S transitions of P(nipam-co-IAM) copolymers appear may depend on polymer composition, molecular weight, ionic strength, pH, and tacticity. Further study on the S-I-S transition and the application thereof for P(nipam-co-IAM) copolymers will be conducted. The application of such an S-I-S transition may be considered as follows. IAM has the potential to become a ring structure (shown in Scheme 1), which is a good chelated structure, and agent. –COOH and –NH_2_ can easily form a chelated structure with metal ions. The characteristics of LCST (S-I-S transition) may capture metal ions by the chelated structure easily at the condition where the copolymer is completely soluble in aqueous solution. Then, when the temperature is changed to be above the LCST temperature, the copolymer becomes insoluble in the solution and precipitates will be filtered out from the solution so that the removal of the metal chelated compound from the solution can be achieved. Further investigation should be also conducted to verify the micelle formation of P(nipam-co-IAM) copolymers.

## Figures and Tables

**Table 1:** Properties of P(nipam-co-IAM) by reversible addition-fragmentation chain-transfer (RAFT).

Sample ID	Lewis AcidXX [Y(OTf)_3_]_0_	Tacticity/%		Mn/10^4^	M_w_/M_n_	Particle Diameter/nm
	Conc./M (mol L^−1^)	m	r			In 25 °C
P_noLA	-	49	51	11.2	1.48	155.3 ± 7.9
I_8 noLA	-	48	52	4.01	1.91	216.2 ± 8.7
I_12 noLA	-	48	52	3.77	1.99	268.4 ± 44.3
P Y	6 × 10^−3^	53	47	9.16	1.33	71.0 ± 1.8
I_8 Y	6 × 10^−3^	52	48	3.11	1.86	332.2 ± 19.6
I_12 Y	6 × 10^−3^	53	47	3.05	1.87	402.3 ± 28.1
